# Progress in Diseases of the Nervous System

**Published:** 1901-06-29

**Authors:** 


### PROGRESS IN DISEASES OF THE NERVOUS SYSTEM.

(*Continued from page* 199.)

**Syphilitic Polyneuritis.**—Cestan[4] has collected several cases of polyneuritis due to syphilis. The condition appears during the secondary stage, at a date varying from one month to thirteen or fourteen months. Several types may be distinguished. In some cases the form is a subacute sensory-motor with pains—spontaneous or elicited—in muscular groups, numbness of the extremities, loss of tendon reflexes, muscular wasting, but no sphincter difficulty or mental impairment. There is also a type which imitates tabes, there being some paresis and sensory alteration of the lower limbs, Romberg's sign and ataxic gait, all subsiding under antisyphilitic treatment. There is lastly a motor form in which sensory symptoms are absent, whilst the muscles waste and the tendon jerks are abolished. In all three types the prognosis is favourable. The facial nerves do not seem to be affected. From the point of view of diagnosis the only difficulty may be offered by some forms of malignant syphilitic affections of the central nervous system in which the prognosis is graver. The use of iodo-mercurial treatment alone will serve to distinguish syphilitic neuritis from the alcoholic or metallic forms.

**Facial Paralysis.**—Bernhardt[5] finds that in isolated cases of facial paralysis, on electrical stimulation of both the paralysed and the unparalysed side, so-called reflex movements occur on the unstimulated half of the face. This may occur both in bulbar lesions and in peripheral facial paralysis. The mode of origin is not always clear; in some it is clearly not reflex but due to diffusion of the current. In other cases it is owing to the passage of healthy muscle fibres from the unaffected to the paralysed side. The collateral symptoms of facial paralysis have been studied by Köster,[6] who concludes that the absence of all other loss of function except derangement in sweating and motor paralysis is indicative of the peripheral seat of a facial palsy below the stylo-mastoid foramen and up to its giving off the chorda tympani nerve. If the interruption is situated at any point above the giving off of the chorda there is not only facial palsy and sudoriferous disturbance, but also derangement in the sense of taste and often also in the secretion of saliva. If the lesion involves the vicinity of the geniculate ganglion there will constantly be, in addition, derangement in the secretion of tears. If the morbid process is above that point, the foregoing symptoms are present except the loss of taste. When the lesion involves the facial nucleus the symptoms will be the same as those mentioned last, and the diagnosis will have to be based on the presence of other symptoms indicative of bulbar disturbance.

**Plantar Reflex.**—Finizio[7] has made a study of the plantar reflex in new-born infants. He finds that in 5 per cent. of the cases it was absent, in 10 per cent. it was indefinite, in 15 per cent. extension of the great toe, or of all the toes, was produced, and in 70 per cent. flexion of the toes resulted. He concludes that in the great majority of new-born infants flexion of the toes is produced. The cases of extension were generally those in which the birth had been abnormal—for example delayed labour, forceps application, face presentation. The plantar reflex is one of the most constant of the skin reflexes; on the other hand the cremasteric reflex is only met with in 2 per cent. of new-born infants; the patellar reflex is, like the plantar, found in 70 per cent. of the cases.

**Apomorphine as a Hypnotic.**—Douglas[8] points out that whilst, as is well known, a tenth of a grain of apomorphine injected subcutaneously will produce vomiting, a thirtieth of a grain similarly administered is a prompt and efficient hypnotic. In mild insomnia and in furious delirium it was found to produce sleep within twenty-five minutes. The sleep is refreshing and restful, and no disagreeable after-effects followed. There is no possibility of a drug habit being formed, as it becomes a vigorous emetic if the dose is increased. There are no cumulative effects. The drug is therefore a safe one, deserving a widespread use.

**Methylene Blue as an Analgesic.**—Klemperer[9] has employed methylene blue as an analgesic in cases of sciatica. In eight cases it failed entirely, in six the pains marvellously disappeared in five days, whilst in the remaining 13 cases the pains, though not abolished, were much diminished, so that the patients could sleep at night. Forty to 80 grains were given daily. The drug is also very useful in tabes, neuralgia, and myalgia. The drug must be used absolutely pure, else gastric, toxic, or diarrhœic troubles ensue. The patient should be warned of the change in the colour of the urine. The action of the methylene blue causes, first, a numbness, which passes gradually into analgesia; its action is rapid, but not of very long duration.

[4] Nouv. Iconograph de la Salpetrière, 1900. [5] Berliner Klin. Woch., 1900. [6] Deutsches Archiv. für Klin. Med., Bd. 68. [7] Pediatria, vol. viii., 254. [8] Merck's Archiv., 1900. [9] Méd. Mod., 1900.

